# Hypoxia-Like Signatures Induced by BCR-ABL Potentially Alter the Glutamine Uptake for Maintaining Oxidative Phosphorylation

**DOI:** 10.1371/journal.pone.0153226

**Published:** 2016-04-07

**Authors:** Pallavi Sontakke, Katarzyna M. Koczula, Jennifer Jaques, Albertus T. J. Wierenga, Annet Z. Brouwers-Vos, Maurien Pruis, Ulrich L. Günther, Edo Vellenga, Jan Jacob Schuringa

**Affiliations:** 1 Department of Experimental Hematology, Cancer Research Center Groningen, University Medical Center Groningen, University of Groningen, Groningen, The Netherlands; 2 Institute of Cancer and Genomic Sciences, University of Birmingham, Birmingham, United Kingdom; 3 Department of Laboratory Medicine, University Medical Center Groningen, University of Groningen, Groningen, The Netherlands; B.C. Cancer Agency, CANADA

## Abstract

The Warburg effect is probably the most prominent metabolic feature of cancer cells, although little is known about the underlying mechanisms and consequences. Here, we set out to study these features in detail in a number of leukemia backgrounds. The transcriptomes of human CB CD34^+^ cells transduced with various oncogenes, including BCR-ABL, MLL-AF9, FLT3-ITD, NUP98-HOXA9, STAT5A and KRAS^G12V^ were analyzed in detail. Our data indicate that in particular BCR-ABL, KRAS^G12V^ and STAT5 could impose hypoxic signaling under normoxic conditions. This coincided with an upregulation of glucose importers SLC2A1/3, hexokinases and HIF1 and 2. NMR-based metabolic profiling was performed in CB CD34^+^ cells transduced with BCR-ABL versus controls, both cultured under normoxia and hypoxia. Lactate and pyruvate levels were increased in BCR-ABL-expressing cells even under normoxia, coinciding with enhanced glutaminolysis which occurred in an HIF1/2-dependent manner. Expression of the glutamine importer SLC1A5 was increased in BCR-ABL^+^ cells, coinciding with an increased susceptibility to the glutaminase inhibitor BPTES. Oxygen consumption rates also decreased upon BPTES treatment, indicating a glutamine dependency for oxidative phosphorylation. The current study suggests that BCR-ABL-positive cancer cells make use of enhanced glutamine metabolism to maintain TCA cell cycle activity in glycolytic cells.

## Introduction

Chronic myeloid leukemia (CML) is a hematological stem cell malignancy mediated by the BCR-ABL translocation between chromosome 9 and 22, t(9;22)(q34;q11) resulting in the Philadelphia chromosome in multipotent hematopoietic stem cells (HSC) [1–3]. The chimeric *BCR-ABL* gene encodes for the constitutively active tyrosine kinase oncoprotein BCR-ABL, which remains in the cytoplasm and can activate distinct intracellular signaling pathways that confer impaired differentiation, enhanced survival and a proliferative advantage [4–9]. Moreover, the BCR-ABL oncoprotein mimics growth factor signaling pathways that can promote cell proliferation further regulating cell metabolism [10]. Yet, little is known about the mechanisms by which BCR-ABL signaling impacts on bioenergetic and biosynthetic needs of cancer cells.

Hypoxia inducible factors 1/2α (HIF1/2) act as transcription factors that are stabilized under hypoxic conditions. HIF1 has been characterized as an important factor controlling cellular metabolism, while the role of HIF2 remains less clear [11,12]. Previously, we identified HIF2 as a downstream target of STAT5 and observed elevated glucose uptake in STAT5 activated HSCs [13]. Several genes associated with glucose metabolism were upregulated by STAT5 in an HIF2-dependent manner, including SLC2A1 and GYS2 [13]. Under hypoxia, it has been shown that HIF1 regulates pyruvate dehydrogenase kinase (Pdk2/4) thus shunting entry of pyruvate into the tricarboxylic acid cycle (TCA), resulting in enhanced lactate production in quiescent HSCs [14]. Furthermore, Yu et al. have shown that a PTEN-like mitochondrial phosphatase, Ptpmt1 primes the switch to mitochondrial oxidative phosphorylation to support the energy demands in differentiating HSCs [15]. These studies highlight two distinct metabolic programs in quiescent and actively cycling normal HSCs. Apart from normal HSCs, HIF1 and HIF2 has also been associated with survival maintenance of primary AML and CML leukemic stem cells (LSCs) [16–18]. Recently, imatinib resistance in BCR-ABL cells was correlated with increased expression of HIF1 which resulted in metabolic reprogramming by increasing glycolysis at the expense of a reduced glucose flux in the TCA pathway and pentose phosphate pathway (PPP) [19]. However, in general it has been proposed that imatinib might not completely block the *de novo* synthesis of purines and pyrimidines or fatty-acid synthesis needed for actively proliferating cells [20,21]. Also, it is plausible that CML cells can still be dependent on alternative sources of energy as well besides glucose.

Glutamine, being the most abundant amino acid in the human plasma, has been shown to be essential for boosting mitochondrial metabolism in c-MYC transformed or *IDH* mutant AML [22–24]. Intriguingly, Le et al. have shown glucose-independent mitochondrial oxidative phosphorylation under hypoxic conditions in the presence of glutamine in P493 cells, a human B cell Burkitt lymphoma cell-line [25]. Hence, apart from being the obligate nitrogen donor for purine and pyrimidine synthesis, glutamine might play an important role in anaplerosis if it is either oxidized to form succinate or if it follows reductive carboxylation for generating citrate under hypoxia [26,27].

In the current study, we combined transcriptome and metabolome profiling in order to understand how oncogenes would impact on the metabolism of leukemic cells. Our data indicate that BCR-ABL could impose hypoxic signaling under normoxic conditions, coinciding with an upreglation of glucose importers SLC2A1/3, hexokinases and HIF 1 and 2. NMR-based metabolic profiling revealed a strong upregulation of lactate and pyruvate in BCR-ABL expressing cells even under normoxia, coinciding with enhanced glutaminolysis which occurred in a HIF1/2-dependent manner via enhanced glutamine import. The current study suggests that BCR-ABL-positive cancer cells make use of enhanced glutamine metabolism to maintain TCA cell cycle activity in glycolytic cells.

## Materials and Methods

### Primary cell isolations

Neonatal cord blood (CB) was obtained from healthy full-term pregnancies after informed consent in accordance with the Declaration of Helsinki from the obstetrics departments of the University Medical Centre Groningen (UMCG) and Martini Hospital Groningen, Groningen, The Netherlands. Donors were informed about procedures and studies were performed with CB by an information sheet that was read and signed by the donor, in line with regulations of the Medical Ethical Committee of the UMCG. All protocols were approved by the Medical Ethical Committee of the UMCG. After ficoll separation of mononuclear cells, CD34^+^ cells were enriched using a magnetically activated cell-sorting CD34 progenitor kit or automatically by using auto Macs (Miltenyi Biotech) as described previously and cryopreserved until further use. Chronic phase (CP) (n = 6) and Blast Crisis (BC) chronic myeloid leukemia (BC CML) blasts (n = 1, #1) from the peripheral blood cells from untreated patients with the t(9;22) translocation, as well as an AML sample (n = 1, #2, FLT3-ITD, NPMwt, IDH1 R132H, t(3;5), +8[10]) were obtained and studied after informed consent in accordance with the Declaration of Helsinki, and the protocol was approved by the Medical Ethical Committee of the UMCG. Donors were informed about procedures and studies performed with AML cells by an information sheet that was read and signed by the donor, in line with regulations of the Medical Ethical Committee of the UMCG. In all cases, mononuclear cells were isolated by density gradient centrifugation and CD34^+^ cells were stained using CD34-PE antibody (BD Biosciences) and selected by sorting on a MoFLo (DakoCytomation) and used in coculture experiments. AML and CP/BC CML co-cultures were expanded in Gartner’s medium supplemented with 20 ng/mL interleukin 3 (IL-3; Gist-Brocades), granulocyte-colony stimulating factor (G-CSF; Rhone-Poulenc Rorer) and thrombopoietin (TPO; Kirin).

### Retroviral and lentiviral transductions

The murine stem cell virus (MSCV)-BCR-ABL-internal ribosomal entry site (IRES2)- truncated nerve growth factor receptor (tNGFR) retroviral vector was cloned into MiNR1 as previously described [28]. Transduction of CB CD34^+^ cells was performed and transduced cells stained with anti-NGFR phycoerythrin (PE) antibody (Becton Dickinson) for analysis as described before [29]. For shRNA silencing, a lentiviral vector expressing a short hairpin against HIF1a was made by cloning the hairpin sequence from pSuper-puro-HIF1a1470 (which was a kind gift from Daniel Chung, Massachusetts general hospital, Boston, Massachusetts) into the pLVUT vector [30]. A short hairpin sequence against HIF2a was constructed by cloning the hairpin sequence from pRetro-Super-HIF2a (obtained from Addgene, addgene number 22100) into the pLVUT vector. A control vector was made by cloning a hairpin against firefly luciferase into the pLVUT vector. Viral particles were generated and lentiviral transductions were performed as described before [31].

### Cell culture and treatment

CB CD34^+^ transduced BCR-ABL positive cells with/without shHIF1 and shHIF2, normal CB CD34^+^ cells as well as K562 cell-lines (obtained from the American Type Culture Collection ATCC, Manassas, USA) cultured at density of 0.3 x 10^6^ cells per ml in 24 wells tissue culture plate for 30–50 days under normoxia (21% O_2_) either in HPGM with c-KIT, Flt3, TPO ligand for primary CB CD34^+^ cells or in IMDM along with 10% FCS and 1% PS for cell-lines. Day 19 CB CD34^+^ as well as BCR-ABL CB CD34^+^ cells were cultured under hypoxia (1% O_2_) for 24 hours and used for NMR analyses as described below. For BPTES sensitivity studies, CB, CB BCR-ABL as well as K562 cells were treated with DMSO control and 20 μM and 40 μM of BPTES and further used for Seahorse analyses.

### RNA isolation and real-time PCR

Total RNA was isolated using the RNeasy Mini kit (Qiagen, Venlo, The Netherlands) according to the manufacturer’s instructions. The real-time RT-PCR was performed using the iScriptcDNA synthesis kit (Bio-Rad, Veenendaal, The Netherlands) with 0.5–1 μg of total RNA and qPCR performed using SsoAdvanced SYBR green supermix (Bio-Rad) in a CFX Connect thermocycler (Bio-Rad). Primers used and optimized PCR conditions were used as mentioned before [31]. Ribosomal protein like (RPL) 27 expression levels were used to calculate relative expression levels and data was analyzed using CFX software (Bio-Rad).

### Transcriptome analyses

Most transcriptome data used in this study was published previously including CB CD34^+^ cells transduced with MLL-AF9 [32], FLT3-ITD [33], NUP98-HOXA9 [34], STAT5A [30,35–37] and KRAS^G12V^ [38] while new data was generated for CB CD34^+^ cells transduced with BCR-ABL which is included in S1 Table. Genome-wide expression analysis was performed on Illumina (Illumina, Inc., San Diego, CA) BeadChip Arrays (Illumina HumanHT-12 v4 Expression Beadchips (47K probesets)). Typically, 0.75 μg of cRNA from two independent transductions was combined and used in labelling reactions and hybridization with the arrays according to the manufacturer’s instructions, and two independent experiments were performed. Data was analyzed using GenomeStudio V2011.1 Gene Expression Module v1.9.0 (Illumina, Inc.) and Genespring (Agilent, Amstelveen, The Netherlands) and is quantile normalized log2 transformed data is provided in S1 Table.

### FACS analyses

CD271 (C40-1457) was obtained from Beckton Dickinson (BD) Biosciences (Breda, The Netherlands). Cells were incubated with antibody at 4°C for 30 min. For blocking nonspecific binding to Fc receptors, cells were blocked with mouse and human anti-Fc antibodies for 10 min at 4°C. All FACS analyses were performed on a FACS Calibur (Becton Dickinson) and data was analyzed using Flow Jo (Tree Star, Inc.). Cells were sorted on a Mo Flo (Beckman Coulter).

### Extraction of polar metabolites and NMR spectroscopy

CB or CB transduced BCR-ABL day 20 cells (+/- 24 hours of hypoxia) were used for NMR analyses. 20–30 million cells were quenched and the intracellular metabolites were extracted, evaporated using a SpeedVac concentrator and stored at 80°C until further analysis. For the NMR analysis dried samples were resuspended in 60 ml of 100mM sodium phosphate buffer containing 500 mM TMSP ((3-trimethylsilyl)propionic-(2,2,3,3-d4)-acid sodium salt) and 10% D_2_O, pH 7.0. Samples were vortexed, sonicated and centrifuged briefly, before being transferred into a 1.7mm NMR tube using an automated Gilson sample handler. One-dimensional 1D ^1^H-NMR spectra were acquired using a 600-MHz Bruker Avance III spectrometer (Bruker Biospin) with a TCI 1.7mm z-PFG cryogenic probe at 300 K. Each sample was automatically tuned, matched and then shimmed before acquisition of the spectrum. Spectra were processed using the MATLAB-based MetaboLab software [39]. The chemical shift was calibrated by referencing the TMSP signal to 0 ppm. Spectra were exported into Bruker format for metabolite identification and to determine concentrations using the Chenomx 7.0 software. The extracellular metabolites were measured directly in the used culture medium. All data presented here are in μMolar concentrations.

### Western blot

For antigen detection, sample preparation, SDS electrophoresis and transfer was performed as described before [31]. The PVDF-FL membrane (Millipore, EttenLeur, The Netherlands) was blocked in odyssey blocking buffer (Westburg, Leusden, The Netherlands) and further probed with SLC1A5 (D7C12, Cell Signaling, Bioké, Leiden, The Netherlands) and β-actin (sc-47778, Santa Cruz Biotechnology, CA) as loading control primary antibody and further detected with Alexa680 and IRDye800 fluorescent conjugated secondary antibodies (Invitrogen, Breda, the Netherlands). The membrane was visualized using odyssey infrared scanner (Li-Cor Biosciences, Lincoln, NE, USA). Signal intensities were quantified using Image studio Lite software (Li-Cor Biosciences, Lincoln, NE, USA) and calculated relative to loading control intensity for each sample.

### Seahorse analyses

K562 cells were maintained in IMDM growth medium with 10% FCS and 1% Penicillin and streptomycin and day 20 cells were used for extracellular flux analyses by seahorse. About 0.1–0.15 million cells were seeded per well in poly-L-lysine (Sigma-Aldrich) coated XF-24 well cell-culture microplates in XF Assay media supplemented with 0.1 g/L of glucose (Sigma-Aldrich) and 0.5 mM of sodium pyruvate (Gibco). The cellular oxygen consumption rate (OCR) and extracellular acidification rate (ECAR) were obtained using an XF24-3 analyzer and XF24 FluxPak from Seahorse Bioscience. The measurement of OCR was performed using XF cell Mito stress kit according to manufacturer’s instructions. Data was analyzed using Wave (Seahorse bioscience) software.

### Statistical analyses

All statistical analyses was performed using the student t test (unpaired, two-tailed) and were expressed as means ± SEM for all other comparisons. Differences were considered statistically significant at p ≤0.05.

## Results

### BCR-ABL imposes hypoxia-like transcriptome changes in human CD34^+^ cells under normoxic conditions

**Fig 1:**
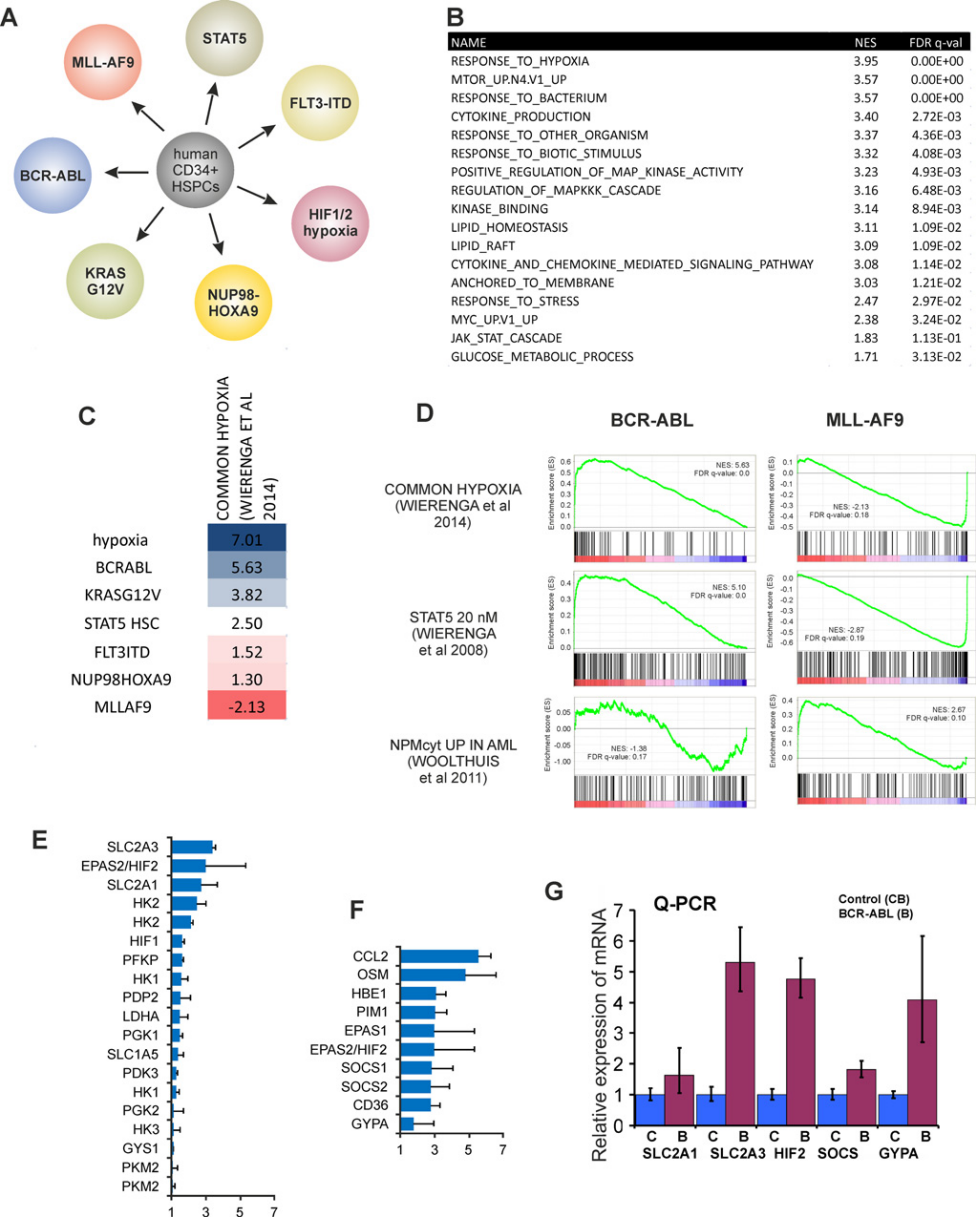
BCR-ABL imposes hypoxia-like transcriptome changes in human CD34^+^ cells under normoxic conditions. **A,** Various oncogene-models in human CB CD34^+^ cells have been generated and genome-wide transcriptome analyses was performed. **B,** GSEA of the BCR-ABL transcriptome data using Broad sets GO and ONCOGENIC SIGNATURES. Highest scoring term was ‘response to hypoxia’. **C,** GSEA of transcriptomes of various oncogene models using a common hypoxia signature we defined previously in CB CD34^+^ cells [25]. NES scores are shown. All FDR q-values are significant and below 0.25, except for NUP98-HOXA9. **D,** GSEA plots indicating that BCR-ABL but not MLL-AF9 cells are enriched for hypoxia and STAT5 signatures, while MLL-AF9 cells are enriched for NPMcyt signatures, as expected. **E,** Upregulated metabolism genes in BCR-ABL cells as determined by Illumina Bead Array. **F,** Upregulated STAT5 target genes in BCR-ABL cells as determined by Illumina Bead Array. **G,** Validation of Illumina Bead Array data by Q-PCRs.

In order to study mechanisms by which oncogenes impact on the metabolism of leukemic cells we transduced human CB CD34^+^ cells with BCR-ABL and performed a genome wide transcriptome analysis. Data were compared to transcriptomes of various other human leukemia models we had generated over the years, including CB CD34^+^ cells transduced with MLL-AF9 [32], FLT3-ITD [40], NUP98-HOXA9 [34], STAT5A [13,30,36,41] and KRAS^G12V^ [38] (Fig 1A, S1 Table). Gene Set Enrichment Analysis (GSEA) was performed on BCR-ABL-transduced CB CD34^+^ cells which revealed, as expected, a strong enrichment for STAT5 and MYC signatures. Interestingly, we also found strong enrichment for hypoxia, response to stress and glucose metabolism gene signatures (Fig 1B). Since we observed an enrichment for hypoxia signatures, data were also compared to transcriptomes of CB CD34^+^ cells grown under hypoxia, or CB CD34^+^ cells expressing activated HIF1A^P402A,P564A^ HIF2A^P405A,P531A^ [31]. Indeed, a strong enrichment was observed in BCR-ABL expressing cells for a common hypoxia signature we identified previously [31] (Fig 1C and 1D). We then compared NES scores for enrichment of our common hypoxia signature in various oncogene models, which showed the highest enrichment in BCR-ABL, KRAS and STAT5 expressing cells, poor enrichment in NUP98-HOXA9 expressing cells, and even a negative enrichment in MLL-AF9 expressing cells (Fig 1C). Some GSEA plots are shown in Fig 1D, highlighting that BCR-ABL but not MLL-AF9 expressing cells showed enrichment for hypoxia and STAT5 signatures. NUP98-HOXA9 and MLL-AF9 share a common HOXA-MEIS1-PBX signature that is also seen in NPMcytleukemias [42–44]. Indeed, we observed that MLL-AF9 cells, but not BCR-ABL cells, were enriched for NPMcyt signatures from primary patient AML samples [44] (Fig 1D). Together, these data suggest that there is a strong variation in the level at which oncogenes can impose hypoxia-like signaling on cells, possibly suggesting a stronger dependency of BCR-ABL cells on intrinsic hypoxic signaling as compared to other oncogene models.

Many glucose metabolism related genes like SLC2A3, SLC2A1, HIF1, HIF2 (Fig 1E) and STAT5 target genes like CCL2, OSM, PIM1, SOCS1 and SOCS2 (Fig 1F) were upregulated in BCR-ABL cells as determined by IlluminaBeadArrays. Upregulation of SLC2A1, SLC2A3, HIF2, SOCS and GYPA was further validated by performing real-time QPCRs on control and BCR-ABL cells (Fig 1G). In summary, these data suggested that BCR-ABL imposes hypoxic signaling under normoxic conditions.

### Knockdown of HIF1 or HIF2 in the absence of bone marrow stroma leads to a proliferative disadvantage in BCR-ABL stem/progenitor cells

**Fig 2:**
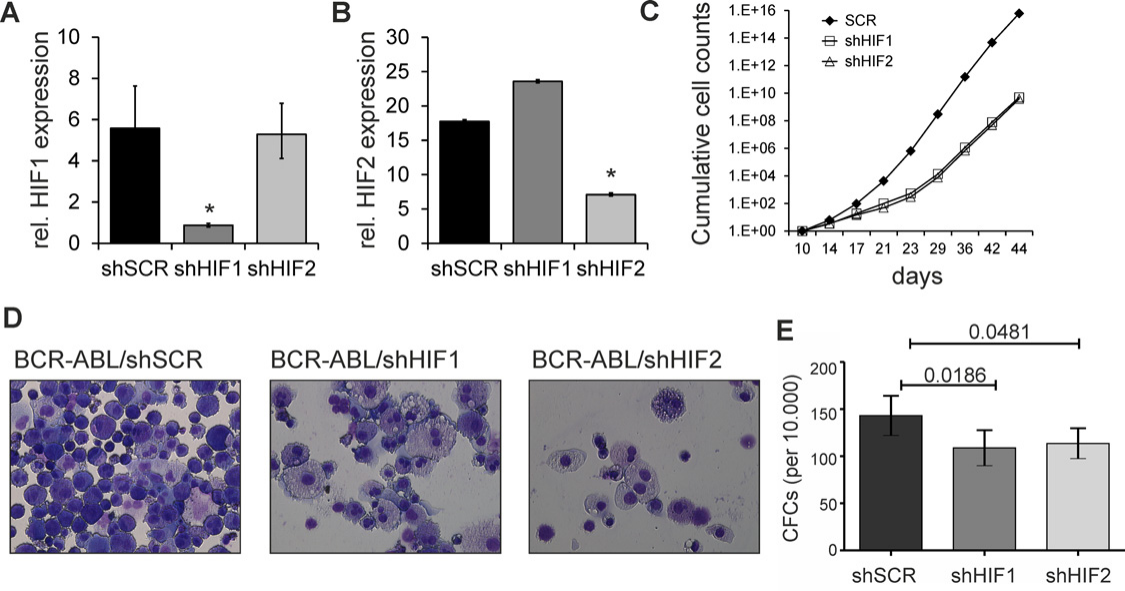
Functional relevance of HIF1 and HIF2 in BCR-ABL stem/progenitor cells grown under liquid culture conditions. **A-B,** Down-regulation of HIF1 and HIF2 by respective short hairpins in BCR-ABL positive cells as compared to scrambled controlwas validated by q-PCR. **C,** Knockdown of HIF1 or HIF2 impaired growth of BCR-ABL positive cells under liquid culture conditions. Data shown is representative of n = 3. **D,** MGG stainings at day 44 (63X magnification). E. Methylcellulose assay indicated reduced colony forming ability of BCR-ABL positive cells upon down-regulation of HIF1 and HIF2 at day 10, p-value<0.05 as indicated in panel.

The hypoxia-induced transcription factors (HIF1 and HIF2) are normally upregulated and stabilized under hypoxic conditions in the bone marrow microenvironment, but we noted upregulation by BCR-ABL under normoxic conditions as well, in line with what we observed previously for STAT5-induced HIF2 upregulation [13]. To determine whether HIFs play an important role in the transformation potential of BCR-ABL expressing stem/progenitor cells we used a lentiviral shRNA approach to down-regulate HIF1 and HIF2 and efficient down-regulation was validated at the RNA level (Fig 2A and 2B). Moreover, HIF1 levels were unaltered in HIF2 down-regulated cells indicating specificity of the short hairpins, while HIF2 levels were slightly increased in HIF1 down-regulated cells suggesting compensatory mechanisms (Fig 2B). Next, cells were expanded in liquid culture conditions driven by SCF, TPO and FLT3L over a period of 30–50 days, and a reduced output was noted upon either loss of HIF1 or HIF2 (Fig 2C). Morphological analyses by MGG staining indicated a more differentiated phenotype at day 44 upon HIF1 and HIF2 down-regulation, while BCR-ABL cells treated with SCR hairpin appeared more immature and blast-like (Fig 2D), in line with what was observed previously [45]. The clonogenic potential of BCR-ABL^+^ cells was also slightly reduced upon HIF1 and HIF2 down-regulation when analyzed by colony forming cell (CFC) assays in methylcellulose (Fig 2E). Together, these data indicate that down-regulation of HIF1 or HIF2 results in considerable impairment in proliferation of BCR-ABL stem/progenitor cells, and also that HIF transcription factors fulfill, at least in part, non-redundant functions in BCR-ABL stem/progenitor cells. Over the time-course of these experiments apoptosis was measured frequently by AnnexinV FACS analyses, but no signs of enhanced apoptosis upon knockdown of HIF1 or HIF2 were observed (data not shown). We also evaluated the role of HIF1 and HIF2 in BCR-ABL expressing cells when grown on a protective bone marrow stromal microenvironment. Intriguingly, HIF1 and HIF2 down regulation had negligible effects on cell the proliferation or colony output when BCR-ABL stem/progenitor cells were cultured on MS5 stroma (data not shown), suggesting that the presence of stromal cells might compensate for the loss of HIF1 or HIF2 by alternative regulatory pathways.

### Hypoxia-induced metabolic changes in BCR-ABL stem/progenitor cells

**Fig 3:**
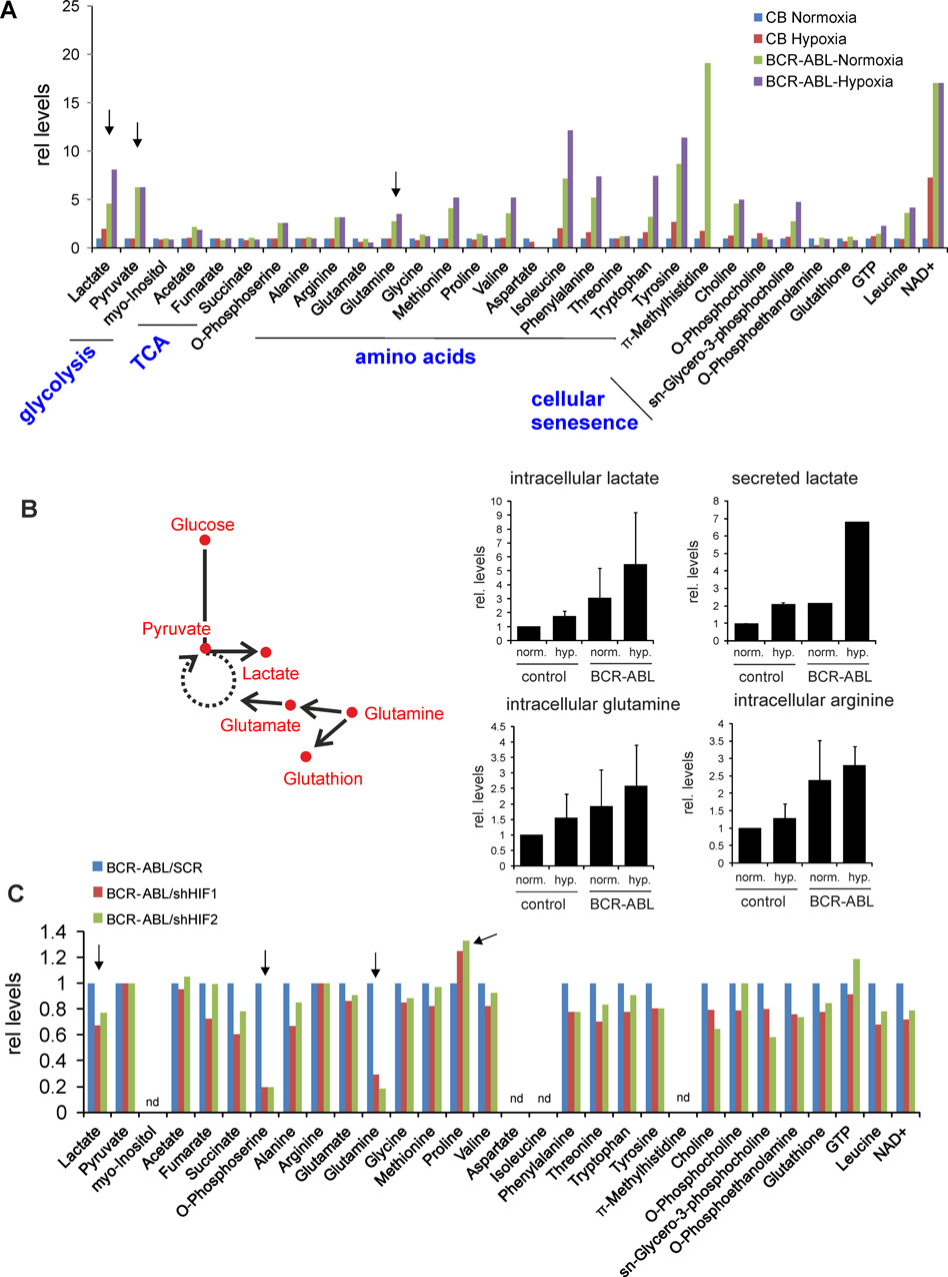
Effect of hypoxia on metabolic changes in BCR-ABL versus normal CB cells as measured by NMR. **A,** Metabolites were quantified using 1D ^1^H-NMR in CB CD34^+^ control cells and transduced CB BCR-ABL cells, cultured under normoxia or placed at hypoxia 24 hrs prior to lyses. Lactate levels were increased in control CB cells grown under hypoxia, and enhanced levels of lactate, pyruvate and glutamate were observed in BCR-ABL^+^ cells even under normoxic conditions. In order to further study glutaminolysis (schematically depicted in **B**) additional experiments were performed to determine intracellular and extracellular levels of several metabolites. Average levels of 2 independent experiments are shown. **C,** 1D ^1^H-NMR analysis showed reduced intracellular levels of lactate and glutamine in BCR-ABL cells upon down-regulation of HIF1 or HIF2.

Next we studied metabolic changes in BCR-ABL^+^ cells cultured under normoxia or hypoxia using 1D ^1^H NMR spectroscopy. We observed striking differences in various metabolites when CB CD34^+^ cells transduced with BCR-ABL were compared to normal control cells grown under normoxia or hypoxia. As expected, BCR-ABL cells exhibited enhanced glycolysis as determined by an increased production of lactate under normoxic conditions which was further increased under hypoxic conditions in comparison to control cells (Fig 3A). Lactate production was also slightly increased in control cells when grown under hypoxia (Fig 3A). Apart from lactate and pyruvate, certain amino acids like glutamine, methionine, valine, isoleucine, phenylalanine, tryptophan, tyrosine and also metabolites related to cellular senescence were increased in BCR-ABL as compared to CB cells under normoxia and hypoxia (Fig 3A). No significant changes were seen in TCA cycle metabolites (Fig 3A).

To confirm these initial data additional independent experiments were performed focusing on glycolysis and glutaminolysis (Fig 3B). Again, increased levels of lactate were observed in BCR-ABL cells compared to control cells under normoxic conditions, which was further enhanced under hypoxia (Fig 3B). We also measured the secretion of lactate, which was increased by BCR-ABL under normoxia as well as under hypoxia (Fig 3B). Furthermore, intracellular glutamine and arginine were increased in BCR-ABL cells, however no particular difference was observed in intracellular glutamate levels in BCR-ABL cells compared to CB cells (Fig 3B). These data suggest that the increase of glutamine in cells reflects rapid glutamine uptake, with a steady-state intermediate concentration of glutamate which is immediately used up by the Krebs cycle, explaining why glutamate levels themselves are not altered. Although the observed differences did not reach statistical significance, probably due to some variation in the absolute concentrations of metabolites that were measured in the individual NMR analyses, the trends were always consistent across multiple independent experiments. Finally, metabolome analyses were repeated in BCR-ABL cells upon knockdown of HIF1 or HIF2. Most notable changes included a decrease in lactate, o-phosphoserine and glutamine levels, as well as increased levels of proline upon knockdown of HIF1 or HIF2 (Fig 3C).

### BCR-ABL cells are dependent on glutamine as an extra source of carbon

**Fig 4:**
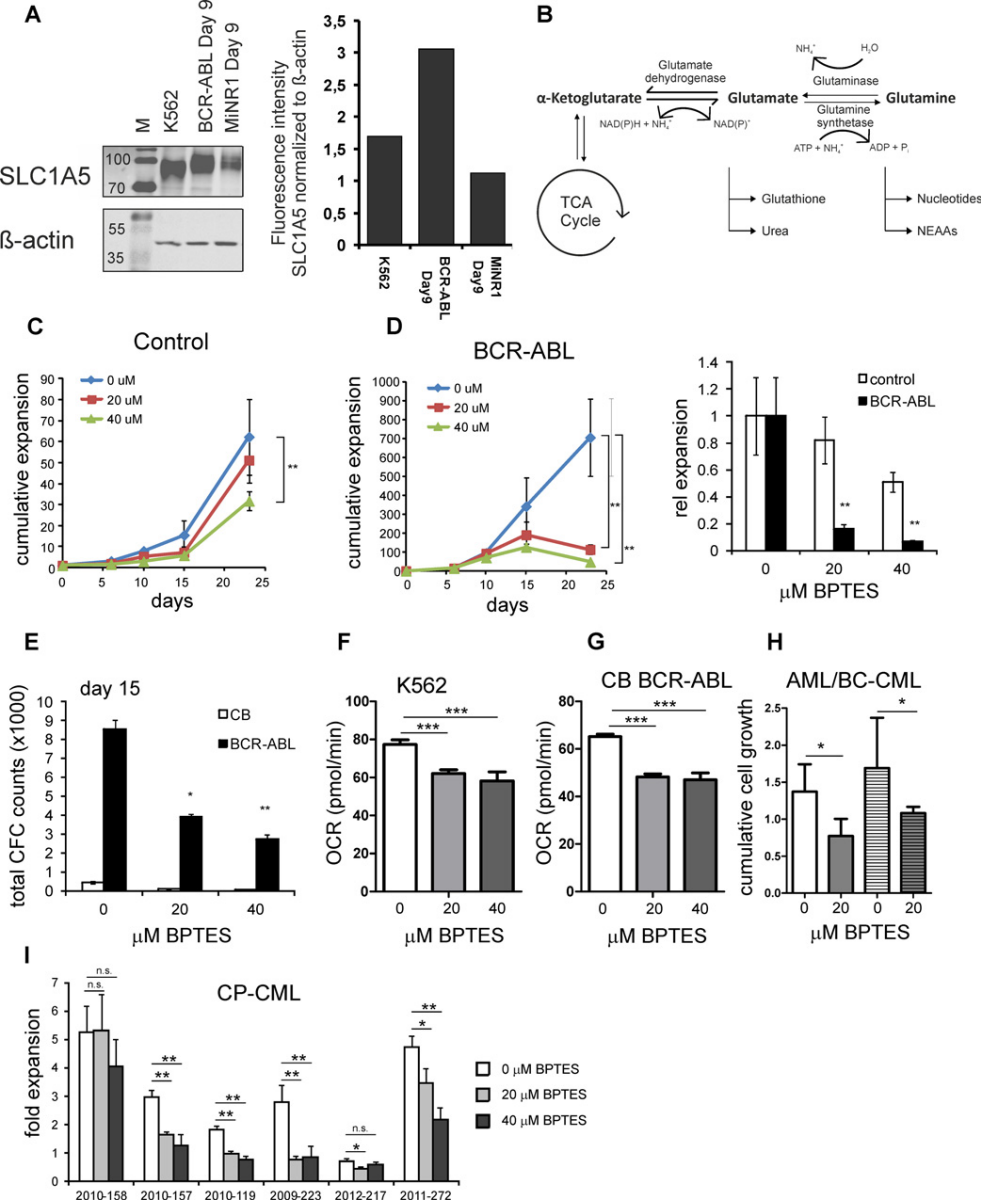
Inihibition of Glutaminase impairs proliferation of BCR-ABL^+^ CB cells. **A,** Enhanced expression of SLC1A5 in K562 and CB BCR-ABL cells as compared to empty vector transduced MiNR1 CB cells. **B,** Schematic representation of glutaminolysis to participate as an extra anaplerotic carbon. **C,** Normal CB cells showed no significant effect with 20 μM of BPTES however higher dose of 40 μM seemed to be toxic. **D,** Dose dependent impaired proliferation observed selectively in BCR-ABL cells as compared to normal CB cells at 20 μM and 40 μM BPTES. **E,** The clonogenic capacity of BCR-ABL cells was compromised in the presence of BPTES, p value: BCR-ABL untreated vs BCR-ABL 20 μM p = 0.0032; BCR-ABL untreated vs BCR-ABL 40 μM p = 0.0117. **F,** OCR measurements in K562 cells treated with BPTES using Seahorse. **G**, OCR measurements in CB CD34^+^ cells transduced with BCR-ABL and treated with BPTES using Seahorse. **H,** Two primary leukemia CD34^+^ patient samples (#1 in open bars, BC-CML sample; and #2 in striped bars, IDH1 mutant AML) were grown on MS5 stroma in the absence or presence of BPTES and cumulative expansion is shown. #1: 0 μM vs 20 μM p = 0.0085; and #2: 0 μM vs 20 μM p = 0.0017. **I**, Six CD34^+^-sorted chronic-phase (CP) CML samples were grown on MS5 stroma in the absence or presence of BPTES. p-values: *<0.05, **<0.01, n.s. = not significant.

The enhanced glutaminolysis observed in BCR-ABL-expressing cells prompted us to study this in more detail. First, the expression of the high affinity importer of glutamine, SLC1A5 (ASCT2) was determined in BCR-ABL cells as compared to normal cells. SLC1A5 was higher expressed in BCR-ABL cells as compared to controls (Fig 4A). These data suggested that enhanced glutamine uptake might be used by BCR-ABL positive cells to convert glutamine to glutamate by glutaminase and finally into α-ketoglutarate to maintain TCA cell cycle activity in glycolytic cells (Fig 4B). We hypothesized that targeting this pathway by BPTES (a glutaminase inhibitor) might provide alternative means to target BCR-ABL positive cells. BCR-ABL expressing stem/progenitor cells were expanded for a period of 3 weeks in the presence of 20 μM and 40 μM BPTES. A dose-dependent inhibition of cell proliferation was evident in BCR-ABL positive cells (Fig 4C). Control cells were also treated with BPTES. Only a negligible non-significant effect of BPTES was observed at 20 μM, while at higher doses of 40 μM a mild but significant reduction in proliferation was seen in normal cells as well (Fig 4D). Furthermore, the clonogenic potential ability of BCR-ABL positive stem/progenitor cells was also compromised in a dose dependent manner upon treatment with BPTES (Fig 4E). Although normal progenitor cells were also decreased upon BPTES treatment these data do point towards an important role for glutamine as alternative carbon source for TCA anaplerosis.

### Treatment with BPTES alters the oxygen consumption rate in BCR-ABL expressing cells

In order to functionally understand the bioenergetic dependency of K562 on glutamine, the mitochondrial respiration rate and glycolysis rate were determined for K562 treated with 20μM and 40μM of glutaminase inhibitor BPTES and compared with rates for untreated K562 over time. The specific changes in oxidative phosphorylation (OXPHOS) as indicated by oxygen consumption rates (OCR) and in glycolysis as indicated by extracellular acidification rate (ECAR) were detected in real-time using the seahorse extracellular flux technology. In line with experiments using CB CD34^+^ cells transduced with BCR-ABL, K562 cells also showed reduced proliferation and dose dependent sensitivity to 20 μM and 40 μM of BPTES (data not shown). Treatment with BPTES had an insignificant effect on ECAR levels in K562 cells indicating no difference in glycolytic rates on BPTES treatment but the observed OCR however was significantly altered even with 20 μM or 40 μM of BPTES (Fig 4F). Similar results were obtained with CB CD34^+^ cells transduced with BCR-ABL upon treatment with BPTES (Fig 4G). These results indicate that OXPHOS is compromised upon BPTES treatment in BCR-ABL expressing cells. Furthermore, glutamine has been suggested to be an important anaplerotic factor for AML with mutations in IDH1/2 [46]. We compared the sensitivity for BPTES treatment of a BC-CML patient sample (#1, open bars) with an IDH1 mutated AML (#2, striped bars). As expected, BPTES significantly suppressed the growth of primary IDH1 mutant AML cells as well as BC-CML cells, suggesting a clear glutamine dependency in both cases (Fig 4H). Furthermore, 6 CP-CML patient samples were analyzed, and 5/6 displayed a significant decreased expansion upon BPTES treatment (Fig 4I).

## Discussion

An enhanced Warburg effect is a prominent feature of many cancers, yet the molecular mechanisms are poorly understood. Alternatively, glutaminolysis enables cancer cells to undergo oxidative metabolism through the TCA cycle. However, a complete understanding of the metabolic alterations due to transcriptional dysregulation via specific oncogenes and tumor suppressors is currently lacking.

Different oncogenes induce different transcriptome changes and thus rely on diverse molecular pathways and signaling networks for propagating leukemia. From this study, we indeed observe that expression of BCR-ABL drives cells into glycolysis, coinciding with an upregulation of various glycolytic enzymes such as glucose importers and hexokinases. Intracellular levels of pyruvate and lactate were increased, and also outside the cells an increase in secreted lactate was detected, even under normoxic conditions. Furthermore, it is evident that BCR-ABL-mediated cell-intrinsic changes induce expression of HIF1 and HIF2 transcription factors under normoxia. We find that BCR-ABL stem/progenitor cells are dependent on HIF signaling in well oxygenated conditions in particular in the absence of a protective bone marrow niche. However, in the presence of bone marrow stromal cells this effect is insignificant, suggesting that the niche might compensate for the loss of HIF1 and HIF2. Furthermore, it appears that MLL-AF9 does not utilize hypoxia-like signaling as much as BCR-ABL-expressing cells do, at least under normoxic conditions. Like MLL-AF9 cells, NUP-98-HOXA9 and NPMc^+^ leukemic cells are characterized by high expression/activation of the HOXA9-MEIS1-PBX axis and cells expressing these oncogenes displayed less overlap with hypoxia/HIF signatures compared to BCR-ABL cells. It is possible that HIF1/2 expression is directly upregulated by BCR-ABL-specific pathways, and indeed especially HIF2 can be directly induced by STAT5 thereby enhancing glycolysis as we described previously [13]. STAT5 is also hyperactivated in FLT3-ITD^+^ AMLs, and CB CD34^+^ cells transduced with FLT3-ITDs also displayed relatively strong overlap with hypoxia signatures. However, most likely alternative pathways apart from STAT5 must play a role as well since HIF1 is upregulated in BCR-ABL expressing cells, but is not induced by STAT5 [13]. STAT5 is not known to be strongly activated by RAS^G12V^, while a significant enrichment with hypoxia signaling was seen in those cells as well, and maybe it is particularly MYC that drives hypoxia-like signaling in these cells. Finally, since both the loss of HIF1 or HIF2 impaired proliferation of BCR-ABL positive cells these data suggest that non-redundant functions of HIF transcription factors might exist, but this is currently unclear and further future studies are required to obtain insight into these differences.

Our studies show that BCR-ABL positive cells also undergo glutaminolysis despite of pronounced glycolysis, suggesting a key role of glutamine as an extra source of carbon in replenishing TCA metabolites. This was evident from increased intracellular glutamine levels in BCR-ABL cells under normoxia as well as under hypoxia. In line with these observations, the glutamine importer SLC1A5 was also overexpressed in BCR-ABL-expressing cells, both at the RNA as well as protein level.

It has been shown that erythroid differentiation also requires glutamine-dependent de novo nucleotide biosynthesis [47]. Others and we have shown that erythroid differentiation is also induced upon retroviral transduction of BCR-ABL [28,48,49], activated STAT5 [35,37,50], or FLT3-ITD [33] in CB CD34^+^ cells, leaving open the possibility that the enhanced glutaminolysis would be, In part, also related to erythroid differentiation. Future studies are required to resolve these issues, but we have also analyzed glutamine dependency in a panel of BCR-ABL^+^ CP-CML and BC-CML patient samples, as well as in an IDH1 R132H-mutant AML sample where this erythroid skewing was not observed. In 5/6 CP-CML samples as well as in the BC-CML sample a significant reduction in proliferation was observed. Interestingly, in IDH1-mutant AML, sensitivity to BPTES was also noted, in line with previously published data [24].

Since we observed that the intracellular glutamine levels were reduced upon HIF down regulation it may suggest that SLC1A5 expression might be under control of these transcription factors, but we have not been able to observe changes in expression of SLC1A5 in HIF1/2-knockdown cells. Possibly, it is the activity of the glutamine importer that might be affected by HIFs, although mechanisms are currently unclear. What is very clear is that conversion of glutamine to glutamate is critically important for BCR-ABL cells since treatment with BPTES strongly impaired growth to a much higher extent compared to normal CB CD34^+^ cells, identifying glutamine as a crucial anaplerotic precursor. The canonical glutaminolysis pathway generates less ATP by converting glutamine to lactate. However, glutamine when oxidized by the TCA cycle can yield 3.5 times more energy as compared to the canonical pathway [25]. Our study indicates no change in lactate levels when treated with 20 μM or 40 μM BPTES at ECAR levels but OCR levels were significantly affected suggesting efficient use of glutamine for effective oxidation. It is thus interesting to note that intact TCA cycling does take place even in the presence of HIFs at normoxia in BCR-ABL stem/progenitor cells. Earlier studies have shown a metabolic shift towards enhanced glycolysis by shunting pyruvate for TCA by inhibition of PDH, essential to maintain HSCs in a quiescent state under hypoxia [14]. It might very well be the case that in BCR-ABL stem/progenitor cells grown under normoxia, the cell cycle is kept under control via similar mechanisms, possibly also involving HIFs, in order to prevent for instance the detrimental effects of a high ROS buildup in the mitochondria and/or oncogene-induced senescence. On the other hand, BCR-ABL cells appear to become even more dependent on glutamine to maintain TCA cycling.

Besides HIFs, we also observe a strong upregulation of MYC by BCR-ABL and it appears likely that alternative pathways driven by cMYC will also be relevant to meet the biosynthetic demands of BCR-ABL stem/progenitor cells. Along with IDH1/2 mutated AML it is intriguing that BC-CML primary cells are also sensitive to BPTES thus exhibiting glutamine dependency even in the presence of glucose (Fig 4H). It will be very interesting in future studies to evaluate whether this addiction can be exploited clinically to treat CML cells with BPTES along with tyrosine kinase inhibitors (TKIs). In conclusion, our combined transcriptome as well as metabolic profiling approach demonstrated that BCR-ABL cells adopt a glucose-dependent glycolysis as well as a glutamine-dependent TCA metabolic profile to fulfill the needs of these leukemic cells.

## Supporting Information

S1 Table This is the S1 Table Transcriptome data used for GSEA. (XLSX)
